# Potential Persistent Borrelia Infection and Response to Antibiotic Therapy; a Clinical Case Study and Review of Recent Literature

**DOI:** 10.3390/antibiotics8040223

**Published:** 2019-11-14

**Authors:** Cozette Moysa, Ross Murtagh, John S. Lambert

**Affiliations:** 1 Independent Researcher, San Juan Capistrano, CA 92675, USA; cozettemoysa@gmail.com; 2 School of Medicine, University College Dublin, D07 A8NN Dublin, Ireland; 3 Infectious Diseases Department, Mater Misericordiae University Hospital, D07 K201 Dublin, Ireland

**Keywords:** borreliosis, tick-borne infections, persistent infections, post-treatment lyme disease syndrome

## Abstract

This report describes the case of an individual who was clinically diagnosed with Lyme borreliosis and initially responded to standard antibiotic therapy. Subsequent to treatment cessation, the patient experienced symptomatic rebound and failed to respond to a second course of the same antibiotic. The patient was eventually diagnosed with both *Borrelia* and *Anaplasma* infections by serological testing performed in a private laboratory. Following a two-month course of combination antibiotic therapy, the patient responded clinically, with a return to almost normal functioning. We discuss this case in the context of recent pre-clinical research examining potential Borrelial persistence despite antibiotic therapy.

## 1. Introduction

Reports of an unusual cluster of children diagnosed with juvenile arthritis and adults with similar symptoms in Lyme, Connecticut and two surrounding towns were brought to the attention of public health authorities in 1975. Many patients reported a previous tick bite and 25% recalled a subsequent rash, often characterized by an expanding center [1]. The causative agent of this mysterious outbreak, initially referred to as Lyme arthritis, was finally identified in 1982. Lyme borreliosis, commonly referred to as Lyme disease, is a vector-borne disease caused by a spirochete of the *Borrelia burgdorferi sensu lato* genus. The genospecies *B. burgdorferi sensu stricto*, *Borrelia afzelii*, and *Borrelia garinii* are primarily responsible for Lyme borreliosis in humans, with the latter two being the predominant pathogens in Europe and the former the primary cause in the United States [2].

The vectors of *B. burgdorferi s.l.* are ticks of the Ixodes genus. In the US, the primary vector is the deer tick *Ixodes scapularis* [2]. Ixodid ticks undergo three developmental stages in their two-year lifespan: larva, nymph, and adult. The tick feeds only once per stage, with the exception of the adult male, which may take sporadic small meals. *B.burgdorferi s.l.* is acquired when the tick, usually a larva or a nymph, ingests a blood meal from an infected reservoir host [3,4]. 

Ixodid ticks undergo the larval stage during the winter and emerge the following spring in the nymphal stage, which is the stage in its lifecycle when the tick is most likely to transmit *Borrelia* infection [4]. The nymphal stage’s significant role as a vector in the north-eastern US results from its small size (< 2 mm), propensity to feed to repletion on humans, and host-seeking activity during the spring and summer months [5,6].

Typical clinical presentation of Lyme borreliosis in the US, where *B.burgdorferi s.s.* is the principal pathogen, is characterized by erythema migrans (EM), often accompanied by malaise, fatigue, headache, arthralgias, myalgias, fever, and regional lymphadenopathy [7]. It is described that in approximately 70–80% of patients, EM is the presenting manifestation, however ~18% or more present with non-specific symptoms and no skin lesion, with a remaining 2–3% presenting with later manifestations such as neuroborreliosis or arthritis [8]. Variable EM rash frequency has been reported in Europe, ranging from 22–60% [9,10,11,12,13,14,15]. Apart from the cases where an EM rash is present, which is diagnostic for disease, guidelines from the Infectious Diseases Society of America (IDSA) require positive serology antibody testing [16]. Independent studies of the accuracy of these tests showing poor sensitivity have been reported by Leeflang et al. [17]. A meta-analysis of commercial tests used in Europe and the USA reported an overall sensitivity of only 59.5% for samples characterized for *Borrelia* infections, and a sensitivity as low as 35.3% for acute/early stage disease [18].

Recent epidemiologic research from the US Centers for Disease Control and Prevention (CDC) reports an estimated incidence of approximately 300,000 cases of Lyme borreliosis annually [19,20]. 

This report illustrates a case of apparent treatment refractory Lyme borreliosis in a returning traveler, with successful resolution of symptoms following a prolonged course of combination antibiotic therapy.

## 2. Case Report

A 58-year-old male Irish resident was traveling in upstate New York in the spring of 2018 and was bitten by a tick on his right thigh with a resultant rash approximately one week later which he identified as an expanding circular and non-pruritic rash. Over the next few weeks, he subsequently developed non-specific symptoms including fatigue, fleeting pains throughout his body, accompanied by difficulties in concentration, which prompted him to attend a general practitioner (GP). Based on his history and presentation, the GP clinically diagnosed the rash as erythema migrans and his condition as *Borrelia* infection and prescribed a three-week course of doxycycline 100mg twice daily. The patient reported feeling much better following receipt of the treatment, but symptoms returned shortly after cessation of the doxycycline.

The patient initially tested negative for Lyme borreliosis by standard Irish enzyme-linked immunosorbent assay (EIA) testing [21]. He was referred to an Infectious Disease (ID) Consultant who stated the patient did not have ongoing active Lyme disease, but at the insistence of the patient, the ID consultant agreed to a further 4-week course of doxycycline twice daily. During this second course of doxycycline treatment the patient did not show any improvement. The patient became progressively more fatigued. He experienced disseminated and migratory arthritis, muscle pains, concentration problems, and reported feelings of pressure in his head. These symptoms caused him to reduce his weekly work schedule by 70 percent. He returned to his GP for further evaluation and testing, four months from onset of symptoms, who repeated laboratory tests.

Repeated Irish Lyme antibody tests revealed B. burgdorferi IgG/IgM C6 EIA positive, but other confirmatory tests (immunoblot) were negative. He was informed he had a false positive Lyme test. He privately obtained TickPlex Plus IgG/IgM antibody testing (registered in Finland), which revealed IgG antibody positive for both *Borrelia* and *Anaplasma*.

He was re-evaluated by another ID specialist in April 2019. Based on medical history and ongoing symptoms and laboratory results, he was started on clarithromycin, rifampicin, and lymecycline (a tetracycline) for suspected ongoing *Borrelia* and *Anaplasma* infections. He had reported a severe allergy to penicillin. At the onset of treatment, the patient had been unwell for one year, with intense fatigue 6/10, distressing abdominal pain 4/10, distressing joint pains 5/10, distressing muscle pains and cramps 4/10, severe neck stiffness and cracking 6/10, stiffness of joints 5/10, intense headaches 6/10, confusion and difficulty thinking 6/10, difficulty with concentrating and reading 3/10, disorientation 4/10, difficulty with speech 3/10, mood swings 4/10, disturbed sleep 7/10, twitching of face and other muscles 3/10, buzzing and ringing in ears 7/10, lightheadedness 5/10, and irritable bladder and urine control problems 6/10. Baseline blood tests revealed normal lymphocyte count but low CD19 percentage, increased immunoglobulins, specifically Ig G antibodies 16.4 (normal range 6–16), normal auto-antibodies, and normal inflammatory markers, CRP and ferritin. He was re-evaluated at the completion of two months of therapy and was found to have complete resolution of most symptoms and a decrease in those remaining. The patient’s specific complaints at the two-month follow-up were ongoing fatigue that had decreased to 3/10, ongoing short-term memory problems 3/10, and disturbed sleep 5/10. He also reported some peri-anal discomfort caused by irritation thought to be secondary to the antibiotic combination. All initial blood test abnormalities returned to within normal limits. He was continued on the combination antibiotic treatment for an additional two months, with a further resolution of symptoms. At re-evaluation two months later, he remained well. After one year of being severely limited in being able to perform normal activities of daily living, the patient was able to return to full-time work after receiving a four-month course of combination antibiotic therapy.

## 3. Discussion

Our case report describes an individual who was clinically diagnosed with Lyme borreliosis and who initially responded to mono-antibiotic therapy. The patient’s symptoms returned, and he failed to respond to a second course of the same antibiotic. He was eventually diagnosed with both *Borrelia* and *Anaplasma* infections by antibody tests performed in a private laboratory. Following a two-month course of combination antibiotic therapy, the patient responded clinically, with a return to almost normal function.

Patients who do not achieve total symptom resolution following initial treatment for Lyme borreliosis are often categorized under the umbrella of post-treatment Lyme disease syndrome (PTLDS). In longitudinal studies of patients with erythema migrans, subjective symptoms such as fatigue, cognitive impairment and musculoskeletal pain have shown to persist for ≥ 6 months after antibiotic therapy in ~10% of patients [22,23]. In some cases, these symptoms may persist for more than 10 years [22]. PTLDS is recognized by guidelines from the IDSA and the United Kingdom’s National Institute for Health and Care Excellence (NICE) [16,24].

The pathogenesis of enduring symptoms remains unclear. Although it is most often recognized as a post-infectious condition, some would suggest that in at least a subset of patients, ongoing symptoms are indicative of persistent infection [25,26,27].

Placebo-controlled, randomized retreatment trials conducted using either parenteral antibiotic therapy alone or followed by a course of oral antibiotics in patients with PTLDS did not show any clinical benefit or showed only modest benefit that would be outweighed by potential adverse effects [28,29,30,31,32]. A reappraisal of retreatment trials published before 2012 was conducted independently by Delong et al. and Fallon et al., each concluding that the results of these trials indicated that retreatment can be beneficial and that they were consistent with potential persistent infection [33,34]. The claims of these reappraisal studies were disputed however by Klempner et al., who argue that neither analysis justifies the conclusion that there is meaningful clinical benefit to be gained from retreatment with parenteral antibiotic therapy [35].

Arguments for persistent infection with *Borrelia* are supported by studies in a diversity of experimentally-infected animal models demonstrating ongoing spirochetal infection despite antibiotic therapy [36,37,38,39,40,41,42,43,44,45]. Additionally, a recent study by Embers et al. demonstrates the presence of persistent, intact, metabolically-active *B. burgdorferi* following treatment of disseminated infection with recommended doses of doxycycline for 28 days in rhesus macaques [46]. Comparable findings in humans also suggest a potential role for bacterial persistence in the symptomatology of PTLDS [25,47,48].

Researchers have noted that patients diagnosed and treated later may develop a refractory arthritic syndrome, which may not respond to the standard recommended therapy [49].

Recent studies have sought to elucidate a plausible pathophysiology for treatment failure and persistent symptoms following antibiotic treatment. Cabello et al. propose that the stringent response mediated by the alarmone (p)ppGpp, a master regulator in *B. burgdorferi*, may be associated with persistence in human hosts and tolerance to otherwise lethal doses of antimicrobials [50]. Pre-clinical studies have demonstrated that a number of the drugs currently recommended to treat Lyme disease, such as amoxicillin and doxycycline, were unable to kill Borrelia burgdorferi persisters in vitro (42,43). Rudenko et al. describe the ability of *Borrelia* persisters to form round bodies, L-form bacteria, microcolonies, or biofilm-like aggregates. These persisters remain viable despite antibiotic therapy, and, in favorable growth environments, are able to reversibly convert into motile forms [27]. Feng et al. show in a mouse model that biofilm-like microcolony and spirochetal and round body variant forms of the stationary phase culture of *B. burgdorferi* were more tolerant to standard Lyme antibiotics and also result in more severe arthritis manifestations than log phase spirochete forms [51].

Coinfections are a significant consideration in patients with Lyme borreliosis. In the US, *I. Scapularis* has been associated with the transmission of other pathogens such as *Anaplasma phagocytophilum*, *Babesia microti*, and *Borrelia miymotoi*. Each of these infectious agents cause nonspecific symptoms, such as headache, myalgia, arthralgia and fatigue, with the fever being generally more prominent than in patients mono-infected with Lyme borreliosis [2].

## 4. Conclusions

Several in vitro drug studies designed over the past few years to test the activity of the “standard” recommended antibiotics against the active and persister cells of *Borrelia* in their stationary growth phase have consistently reported treatment failure. The phenomenon of antibiotic tolerant persister cell formation contributing to treatment failure provides a plausible theory as to why symptoms persist or recur in many patients after repeated trials of antibiotics. In light of these recent findings, the treatment of Lyme disease must not be categorized or dictated to the treating physician as a one-size-fits-all, “cookbook” style of therapy.

Persisters and biofilms are well-recognized as providing additional challenges in treating many other infectious diseases. The recommended treatments for chronic and persistent infections are different and more complicated than the treatments for the same infections when they are treated during the acute phase and the therapeutic window is open. Treatment failures for all infectious diseases are common in medicine. Treatment guidelines do not always adequately treat the individual patient; therefore, individualized treatment regimens are sometimes necessary.

Research must be conducted to identify new agents, or combinations of new and currently available agents, to treat all stages of Lyme borreliosis. We need better diagnostics that use antigen-specific tests to enroll patients in future studies, and antigen-specific tests to measure “test of cure”. Multiple factors contribute to the complexity of these conditions, with infection triggering inflammation and compromising an immune system that is unable to clear tick-borne infections. We need a better understanding of these factors. It appears that ongoing persistent infection triggers a cascade of inflammatory and immunological processes.

Our case report identifies some interesting challenges, including challenges in making a clinical diagnosis, imperfect antibody tests for Lyme, with tests that were called “false positive”, the lack of recognition of “co-infections”, and the challenges of how to manage patients who are still unwell following a standard of short course doxycycline. Recent publications have suggested that persistent symptoms post standard treatment may indeed represent ongoing infection refractory to standard treatment, and the response and cure of our patient with longer treatment and combination antibiotic treatment supports many of the recent publications regarding persistent infection, refractory infections, biofilm, and persistent forms of *Borrelia* and co-infections. As clinicians increasingly recognize these persistent infections, and that indeed such infections can be treated and cured, it will be important to conduct well-designed studies to better understand those patients who would benefit from such a treatment approach.
